# Morpho-Functional Traits in Pura Raza Menorquina Horses: Genetic Parameters and Relationship with Coat Color Variables

**DOI:** 10.3390/ani12182319

**Published:** 2022-09-07

**Authors:** Davinia I. Perdomo-González, Rocío de las Aguas García de Paredes, Mercedes Valera, Ester Bartolomé, María Dolores Gómez

**Affiliations:** 1 Escuela Técnica Superior de Ingeniería Agronómica (ETSIA), Universidad de Sevilla, Carretera de Utrera km1, 41013 Sevilla, Spain; 2 Asociación de Criadores y Propietarios de Caballos de Raza Menorquina, Edificio Sa Roqueta, C/ Bijuters, 36, Bajos, 07760 Ciutadella de Menorca, Spain

**Keywords:** conformation, equine, genetic correlations, heritability, linear type traits

## Abstract

**Simple Summary:**

In this study, we estimated genetic parameters of 46 linear morpho-functional traits, and analyzed the relationship between two coat color traits (quality of black coat color [QB] and the quantity of white marks [WM]) and other linear morpho-functional traits within the breeding program of Pura Raza Menorquina horses, whose studbook only permits the use of black-coated animals with a small quantity of white marks as breeding stock. A total of 772 records from 333 animals were analyzed to estimate genetic parameters for 46 linear traits scored by four appraisers using seven classes. Heritability values for morpho-functional traits were low to medium and matched the range in the bibliography. Medium heritability values were obtained for both coat color traits (0.36 for QB and 0.23 for WM). Genetic correlations between coat and morpho-functional traits ranged between 0.015 and 0.816 in absolute value for QB and between 0.014 and 0.638 in absolute value for WM. The highest correlation values were obtained between QB and upper neck line (0.816) and between WM and form of the hoof (0.638). It was observed that the animal group with low and the group with high breeding values for QB and WM had a clear differentiation of the other mor-pho-functional traits.

**Abstract:**

The studbook of Pura Raza Menorquina horses only permits the use of black-coated animals with a small quantity of white marks as breeding stock. Its breeding program uses linear morpho-functional traits as selection criteria. Our aim was to estimate the genetic parameters of linear morpho-functional traits, and reveal relationship of quality of black coat color (QB) and percentage of white marks (WM) with the other morphological and functional linear traits in this breed. A total of 46 linear traits were scored by four appraisers using seven classes, with a total of 772 records from 333 animals (≥4 years old). Univariate animal models using a Bayesian approach were used, with a pedigree of 757 animals. Sex (two) and appraiser-season (13) were included as fixed effects, age as a linear covariate, and permanent environmental and additive genetic as random effect. The heritabilities of the morpho-functional traits were low to medium (0.09–0.58) and matched the range in the bibliography. Heritabilities for coat color traits were 0.36 for QB and 0.23 for WM. The highest genetic correlations were obtained between QB and upper neck line (0.816) and between WM and form of the hoof (0.638). The negative signs of most of the genetic correlations between WM and the functional traits is also remarkable, contributing to the selection of functional traits against the presence of white marks in this population. A clear genetic differentiation was observed between animals with better breeding values for QB and WM, corroborated by a study on founders. In conclusion, QB and WM could show different genetic backgrounds.

## 1. Introduction

The current conformation of horses is the result of both natural and breeders’ selection, and the traits evaluated for each breed depend on their breeding purposes [1]. Conformation has long been a driving force in horse selection and breed identification, particularly as a predictor of performance and susceptibility to injury [2]. However, it is also important for aesthetics, wellness, and the durability and functionality of horses [3] because conformation defines the limits of the range of movement and function of the horse and its ability to perform [4]. Estimated relative economic values of selection criteria for riding horses, which are based on sale prices, indicate that conformation and movement are the most important traits in horse breeding [5,6] and achieve the highest prices. Conformation traits can be conditioned by several factors (e.g., breed, nutrition, age, or sex) and also coat color [7]. In addition, coat color is one of the most noticeable animal features and has interested and intrigued horse breeders for centuries [8].

The genetic evaluation of conformation traits is of major importance in horse selection because it is a tool for indirect performance selection. This is because the heritability estimates for conformation traits are often higher than those estimated for performance traits. Some authors [9] have affirmed that the efficiency of indirect selection for performance traits depends on genetic variability of the conformation traits and is also related to genetic associations between conformation and functional performance traits [10,11].

The current trend for breeding sport horses combines selection for conformation and performance. Unfortunately, most of the information on sport performance only becomes available later in the horse’s life. For this reason, the selection of riding horses is based primarily on other related traits, which are available at a younger age and have higher heritability values and adequate correlations with the selection traits [9] (such as conformation traits). In this context, to economize money and time, Gómez et al. [2] reco-mmended the use of a two-stage selection program, with a first selection based on the morphological features of the animal and a second selection based on the animal’s performance in equestrian events for Spanish horses. 

The Pura Raza Menorquina horse (PRMe) is an endangered native breed established in 1988. However, they were traditionally bred and located on the island of Menorca (Ba-learic Islands, Spain). Originally, they were mainly used as draught animals in agriculture and forestry. However, it is a highly versatile breed with excellent qualities (beauty, boldness, intelligence, nobility, agility, and resistance) that make it a highly-prized animal whether for dressage or as a saddle horse, a work horse, a light cart-horse or even a sport horse [12]. Nowadays, they are mainly used in Classic and Menorcan Dressage with very good results.

The last official update of the Spanish Ministry of Agriculture database [13] reported a population size of 3798 active individuals, including 624 stallions and 764 mares, of which only 2906 were located in Spain. The remaining 23.5% of the PRMe population is located in foreign countries, contributing to the ex-situ conservation of its genetic diversity, mostly in other European countries such as France, Germany, and Italy. Although some PRMe horses are also located in the USA and Russia. 

The official breeding program of PRMe horses (with conservation and selection aims) and their current uses have probably influenced their body conformation and type, as well as their movements and performance. Thus, Menorcan breeders tend to select animals based on conformation to achieve good performance for Menorcan and Classic dressage [12]. From a morphological point of view, they are a medium-sized breed (1.60 m average height at the withers) with a sleek silhouette and a sub-convex to straight profile. According to breeding regulations, only black animals with a small quantity of white marks can be used as breeding stock [14].

The main objective of this study was to estimate genetic parameters of morphological and functional traits, and analyze relationship of quality of their black coat color (QB) and the percentage of white marks (WM) with the other morphological and functional linear traits.

## 2. Materials and Methods

### 2.1. Materials

For this analysis, we used the linear morpho-functional data of PRMe horses provided by the Asociación de Criadores y Propietarios de Caballos de Raza Menorquina (ACPCRMe). To avoid subjectivity to an ideal type in the comparison, the overall score was replaced by several simple traits, which are linearly evaluated on a scale from one biological extreme to the other; in this way, linear evaluation does not grade an animal, but rather describes it [15].

Linear type traits scored by four skilled horse appraisers with the same evaluation criteria over nine years of evaluation (2013–2021) were considered in the current analysis. A total of 772 records from 333 animals (188 males and 145 females from 118 different studs) were recorded for 46 linear morpho-functional traits in the studs, in sport competitions, and in morphological events held for PRMe horses. Thus, the average number of records per animal was 2.32 and the average number of records per stud was 5.51. The animals were at least four years old, and the average age of the recorded animals was 8.44 years. The animals analyzed represented 12.9% of the population registered in the official Menorca Horse Studbook, which were active and located in Spain in 2021, thereby constituting the nucleus for selection and the majority of the population of PRMe horses all around the world. In this way, the results obtained in this study can be considered descriptive of the current status of this genetic resource and can be used to control its development in future years.

To collect the information, a structured score sheet with a scale of seven categories (from one to seven, without half points) was used by appraisers, in which the extremes represented the biological extremes of the population for the linear traits (see Table 1). Both extremes and the central class were defined on the sheet. The traits can be grouped into two coat color traits (QB and WM), 35 morphological traits (nine for the head and neck region, 14 for the body region, and 12 for the limbs), and nine functional traits evaluated with the horse led by hand at walk (four) and trot (five).

For data collection, the QB was defined using the same criteria as Smith [16], who described two different types of black coat: non-fading (jet black, which is charcoal black with a metallic shine) and fading (black coat color without shine, fading to a reddish-brown tinge). In PRMe horses, the appraisers used seven levels for black coat, according to their quality, which covered the whole range of both types of black coat defined by the authors cited above. The WMs were defined by seven classes using the ranking of penalties established in the official PRMe Horse studbook by their locations and extensiveness.

### 2.2. Methods

Preliminary analyses of variance were carried out using the univariate GLM procedure in SAS software v.9.4 [17] to assess the relative importance of the non-genetic effects that could influence the morpho-functional traits analyzed in PRMe horses. Permanent environment, sex (two levels: male and female), the combination of appraiser and the season (13 levels), quality of the black coat color (grouped into three levels: good [6,7], normal [4,5], and bad [1,2,3] quality), and percentage of white marks (grouped into three le-vels: without WMs [1,2], few WMs [3,4], and many WMs [5,6,7]) were included as fixed effects; the age was also included as a linear covariate. 

The owner stud of the animals was not included in the model because no statistically significant differences were detected for most of the analyzed traits. This could be caused by (1) the similar management systems of the animals in the different studs (females were free in the countryside throughout the year with ad libitum feeding, except when they were close to giving birth, and males were in individual stables with temporal access to a paddock and controlled feeding) and (2) the geographic nearness of the different studs because all of them were located on Menorca island with approximately 700 km^2^ of surface and the same weather conditions.

The heritability values were calculated based on the variance components estimated using univariate animal models with a Bayesian approach via Gibbs sampling using the GIBBSF90+ module of the BLUPF90 software [18]. The following model was fitted to estimate the genetic parameters for all of the traits:

y=1µ+Xb+Zu+Wpe+e

where ***y*** was the vector of observations for a particular trait of the analyzed traits; ***µ*** was the overall mean; **1** is the vector of ones; ***b*** was the vector of the fixed effects (sex, appraiser-season and fixed regression on age at evaluation); ***u*** was the vector of random additive genetic effect; ***pe*** was the vector of random permanent environmental effect of the animal; and **e** was the vector of random residual effect. ***X*** was the incidence matrix relating observations to fixed effects, ***Z*** was the incidence matrix relating observations to additive effects, and ***W*** was the incidence matrix relating observations to random permanent environmental effects.

Also, bivariate models were implemented to estimate the genetic correlations between the two coat color traits (QB and WM) and the other morpho-functional linear traits. The bivariate models fit the same effects as the univariate models.

The Gibbs sampler was run for 1,000,000 rounds, with the first 100,000 considered as burn-in and then every 10th sample saved for later analysis. Posterior means and standard deviations were calculated to obtain estimates of (co) variance components. Convergence of the posterior parameters was assessed by visual inspection of trace plots of posterior distributions generated by the Coda R package [19]. 

The pedigree data for the estimation of the genetic parameters was composed of 757 animals born between 1961 and 2018 (341 males and 416 females), constituting a total of five generations of animals registered in the official Studbook of PRMe horses managed by the ACPCRMe. 

Finally, four subpopulations of animals were created according to their EBVs for each coat trait analyzed. Subpopulation A included animals with an EBV belonging to the better 25% for each trait (quality of the black coat color, A_, and white marks, _A); subpopulation B included the remaining animals with an EBV belonging to the lower 75% (quality of black coat, B_, and white marks, _B). To estimate the EBVs of WMs, a change of the scale was needed because it varied between 1 (without white marks, the desirable option) and 7 (with a lot of white marks, the least desirable option). Finally, ENDOG software [20] was used to estimate the Mahalanobis distances between the four groups (AA, AB, BA, and BB) using the EBVs of all the linear morpho-functional traits included in the analysis, both morphological and functional, to evidence the relationship between the groups.

## 3. Results and Discussion

### 3.1. Descriptive Statistics of the Data

Descriptive statistics of the 46 linear traits analyzed in PRMe horses are reported in Table 2. Coat color traits showed mean values ± standard error ranging between 1.51 ± 0.036 for WM and 4.80 ± 0.046 for QB. For the morphological traits, the mean values ± standard error ranged between 3.21 ± 0.025 for the cannon bone perimeter (trait code 29) and 5.23 ± 0.042 for the head width (3), with a global average of 4.22, which was close to the central value of the scale. For the functional traits, the average values ± standard error ranged between 4.04 ± 0.031 for walking activity (38) and 4.71 ± 0.038 for walking clarity (39), with a global average of 4.46, which was also close to the central value of the scale.

In the morpho-functional evaluation of the PRMe horses, a total of seven classes were used by the appraisers to assess the traits, the same number as was used for morpho-functional evaluations in Thoroughbred horses [4] and for American Quarter Riding horses [21]. Also, the whole classes were used in 47.83% of the linear analyzed traits, being 6 of more classes used in 89.13% of the analyzed traits. Therefore, we can consider that the complete scale was used in the population analyzed, as has occurred in other horse po-pulations (Czech-Moravian Belgian horses and Silesian Noriker [22]; Old Kladrub horses [23,24]; Pura Raza Español horses [25,26]; and sport horses in the Czech Republic [9]). All of the variables met the assumption of a normal distribution.

The coefficient of variation (CV) is considered the most important measure of variation. It generally assumes that the higher the phenotypic variation of traits, the greater the genetic variation, which guarantees a sufficient selection response in populations [23]. The estimated CV in PRMe horses was of a medium–high level, with 26.49% for QB (1) and 64.95% for WM (2). 

For morpho-functional traits, the lowest CV was observed for the upper neckline (10; 15.31%) within the morphological traits and for walking activity (38; 21.42%) within the performance traits, showing that their phenotypic variation is limited biologically in this population. On the other hand, the highest CV was estimated for the form of the withers (13; 32.89%) within the morphological traits and trotting suspension (46; 25.45%) within the performance traits, showing that there is higher phenotypic variation in these traits than in other traits. The CVs obtained were in the range of those reported for linear traits in Dutch Warmblood Riding horses (10.14–26.04% [10]), Heavy Draught horses (11.38–38.54% [1]), Old Kladrub horses (2.39–40.14% [22,23,24]), Pura Raza Español horses (6.94–51.68% [25,26,27]), and sport horses in the Czech Republic (2.91–20.27% [9]). In general, the analyzed population showed sufficient variability.

The influences of the different non-genetic effects are shown in Table S1. All of the analyzed non-genetic effects were statistically significant (*p* < 0.05) for some analyzed traits. Permanent environment, combination of the appraiser and the season, and sex were significant for most of the analyzed traits and were therefore included in the genetic model used in this study. The coefficient of determination (R2) was also estimated for all of the traits, with values higher than and close to 0.55. In this way, the model accounted for over 60% of the variance for most of the linear morpho-functional traits analyzed in the PRMe population.

### 3.2. Genetic Parameters

Coat color is one of the most noticeable animal features and has interested and intrigued breeders for centuries [8]. It is believed that most of the phenotypes currently observed in the modern horse are the result of domestication and selective breeding, and different breeds exist that are mainly defined by the color and patterns of their coats as a result of breeders’ selection criteria, such as Paint and Appaloosa horses [28,29]. This is also the case of PRMe horses, in which black is the only coat color authorized for the breeding stock registered in the official population studbook. However, the pattern of white spots is also a major attribute, and it determines breeding practices in the PRMe production system because individuals showing large white marks on the head, limbs, or the rest of the body are barred from being used as breeding stock for the official studbook. 

A horse’s coat color is generally understood as a qualitative trait with Mendelian inheritance. Here, black coat color is determined by a recessive homozygote genotype at the Agouti locus and at least one dominant allele at the EXTENSION locus (aa E-) [30]. However, there are remarkable differences in color phenotypes that are not explainable by Mendelian inheritance. Different authors [16,31] indicated that there are two different types of black coat color (which they termed non-fading and fading black coats), in which the genetic determination is unknown, but age, sex, season, feeding, housing system, and body part are environmental effects that could significantly affect their expression. One plausible reason for this could be that the estimations of heritability for overall coat color reveal the dominant effect of environmental factors on total variability [31].

The heritability values of the 46 linear morpho-functional traits analyzed in the PRMe population are shown in Table 3. Although the data set may seem small, it includes the selection nucleus of the Menorca horse population and the majority of the horses all around the world.

Medium heritability values were obtained for the linear coat color traits, 0.36 for QB (1) and 0.23 for WM (2), which evidenced the level of selection that can be carried out for both linear traits related to the coat color in PRMe horses. These values were in the range of those reported for the quality of black coat color in Old Kladrub horses (0.14–0.37 [16]) and for the white marks in Hucul horses (0.68–0.69 [32]), Lipizzan horses (0.23–0.71 [33]), Swiss Franches-Montagnes horses (0.52–0.69 [34]), and Arabian horses (0.77 [35]).

A previous study [16] postulated that differences in the genetic determination of fa-ding and non-fading black coat color could be influenced by modifying genes with only a minor effect, but which could be cumulative. Different hypotheses about the inheritance of white marks have also been postulated. First, Woolf [35] concluded that complex genetic systems and non-genetic factors determined the presence of common white marks in Arabian horses. Also, Stachurska and Ussing [36] postulated about the polygenic inhe-ritance of white marks that the ultimate extension of markings was influenced by genes, as well as by intrauterine factors. These authors concluded that the high heritability and QTLs involved mean that selection both towards and against white marks is effective. However, the polygenic inheritance makes it impossible to completely eradicate them because the genes affecting white marks might be recessive and marked by dominant genes and may therefore be difficult to identify. The medium heritability values obtained in PRMe horses evidenced that the influence of external factors is also very important in the phenotypic expression of these coat traits.

Besides the existence of possible pleiotropic effects associated with specific coat color and some conformation [7], performance [37], health [38], and temperamental traits [39], our results evidenced the need to analyze the relationship between QB and WM traits in PRMe horses and the linear morpho-functional traits included in this study.

Heritability values estimated with the univariate animal models for the morphological traits obtained in the present study were of low to medium range (0.09–0.58), showing the higher values of 0.58 for head profile (6), 0.44 for head length (4), and 0.41 for thorax depth (16). In general, the heritability values we obtained were in the range of those reported for linear conformation traits in other horse breeds, which ranged between 0.03 and 0.68 (in Italian Heavy Draught Horses [1], Old Kladrub horses [24,40], sport horses in the Czech Republic [9], Belgian Warmblood horses [41], and the Pura Raza Español [7,25]).

The population of PRMe horses analyzed in this study showed similar heritability values to Belgian Warmblood and Pura Raza Español for head-neck junction (0.26 and 0.14–0.23, respectively [25,41]), to the Old Kladrub horse breed and Pura Raza Español for height of the withers (0.17 and 0.19–0.21, respectively [24,25]), to the Belgian Warmblood for shoulder length (0.31 [41]), to Pura Raza Español for loin length (0.10–0.14 [25]), form of back-loin line (0.12–0.19 [25]), and croup length (0.17–0.19 [25]), to Pura Raza Español and the Old Kladrub horse breed for chest width (0.31 [27,40]), to the Old Kladrub horse breed for forelimb side view (0.10 [40]), and to Pura Raza Español horses for hock side view (0.04–0.09 [25]). Also, the shoulder angle slope, the croup angle, and the hock rear view also showed a similar heritability value to a previous study carried out in the PRMe horses’ population (0.10, 0.23, and 0.21, respectively [12]).

The heritability values estimated for the functional traits obtained in the present study were also of low to medium range, with values of 0.09 for walking activity (38) and walking clarity (39), 0.32 for walking suppleness (41), and 0.33 for walking amplitude (40). The values obtained for these kinds of linear traits were lower than those reported in the reviewed bibliography for linear functional traits in other horses, ranging between 0.18 and 0.52 in sport horses in the Czech Republic [9] and Belgian Warmblood horses [41]. Only walking amplitude showed a similar heritability value to Belgian Warmblood horses and trot amplitude was similar to sport horses in the Czech Republic and the PRMe horses analyzed (0.38 [41] and 0.20 [9], respectively).

Genetic parameters such as heritability are influenced by gene frequency, estimation method, statistical model, and trait nature. Since there may have been differences in the factors used in each study, the slight variations in values between the present study and previous studies might be attributable to those differences. The medium to high estimated heritability for the traits in the current study indicates that genetic improvement would be possible in these traits and allow us to foresee the possibility of making good genetic progress through the breeding program.

Understanding the relationships between morphological traits is extremely useful in animal breeding for determining both the breeding criteria and the possible breeding response of selection programs [25]. However, unfavorable correlated responses with other important or economic characters of the breed must also be avoided. Therefore, the correlations between the linear traits need to be estimated before including the morpho-functional traits into the breeding selection programs of these horses.

For several centuries, behavior, conformation, performance, and suitability characteristics of horses have been attributed to coat color in different populations. For example, coat color influences the conformation traits analyzed in Old Kladrub horses [23] and in Pura Raza Español horses [2,7]. In previous analyses on the same breeds, it has also been associated with certain conformation defects such as cresty neck [27] and ewe neck [42] and diseases like vitiligo or melanoma [7]. However, this is not exclusive to these populations. Its effects on horse behavior [43,44,45], temperament [39], and performance [37,46] have also been reported. In addition, there is still a strong belief among horse breeders that these traits account for differences between horses of different colors [47,48].

Table 4 shows the genetic correlations estimated with bivariate models for the coat color traits (QB and WM) and the other 44 morpho-functional traits analyzed in PRMe horses. Estimated genetic correlations had large standard errors as shown by large stan-dard deviation of the posterior samples.

In general, both color traits showed low to high correlations with the 44 morpho-functional traits. The highest values obtained for QB were with upper neck line (10; 0.816), shape of the withers (13; −0.767), cannon bone perimeter (29; −0.738), back length (17; 0.618), and head profile (6; 0.609). The highest correlations estimated for WM were with form of the hoof (33, −0.807), shoulder angle slope (15, 0.638), fore knee perimeter (28, 0.617), fore-hoof front view (34, −0.603), shape of the withers (13, 0.577), buttock length (22, −0.576), and walking clarity (39, −0.574). The existence of an important relationship between the coat color traits and other linear traits could be caused by a pleiotropic effect, which should be analyzed with the adequate tools.

It is important to note that for QB (1), 37.14% of the genetic correlations with morphological traits were negative, and none of them were correlated with functional traits. For WM (2), 48.57% of the phenotypic correlations with morphological traits were negative whereas 88.89% of the correlations with functional traits were negative. However, in the PRMe population, animals with or without a lower percentage of white marks are required as breeding stock. Therefore, these negative correlations are very interesting in the breeding program.

The genetic correlation between both coat color traits (QB and WM) was −0.272. That means that selection to improve the quality of black coat color implies a decrease in the percentage of white marks, as is desired in this population.

**Figure 1:**
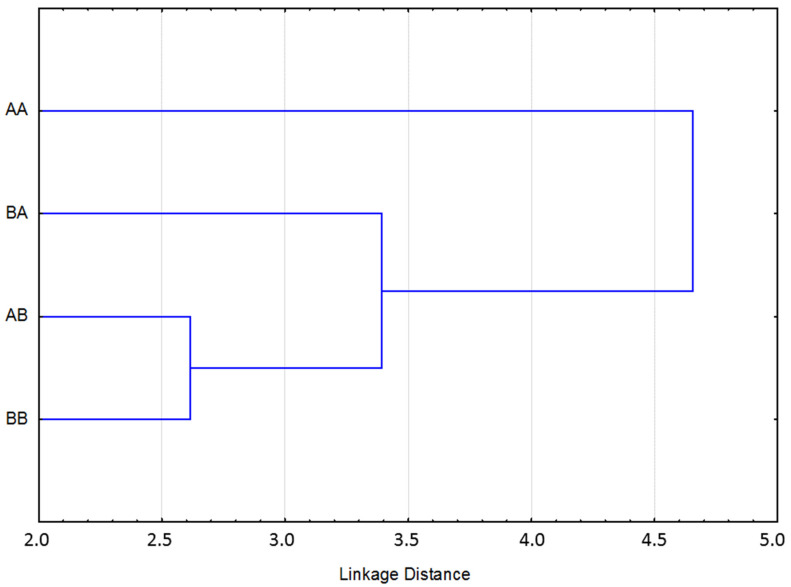
Mahalanobis distances between animals using their breeding values for all the morpho-functional traits analyzed in Pura Raza Menorquina Horses. Animals which have obtained the better 25% breeding value for quality of coat color (A_) and white marks (_A); animals which have obtained the remaining 75% for quality of coat color (B_) and white marks (_B).

Finally, for the graphic representation of Mahalanobis distances, animals were grouped by their EBVs for both traits related to coat color included in the analysis, taking into account all of the morpho-functional linear traits analyzed. The population’s average relatedness was 2.28%, the average inbreeding was 0.6%, and 8.1% of all the pedigreed animals presented some level of inbreeding. The results are shown in Figure 1, where individuals showing the 25% best EBVs for QB color (A_) and WM (_A) were compared with the remaining 75% for the same traits (B_ and _B, respectively). A clear differentiation can be observed between animals included in the group of individuals with the top 25% of EBVs for QB and WM (AA) and the other analyzed groups because they were represented in clearly separated clusters. Also, a clear relationship can be observed between animals included in the groups of individuals in the remaining 75% for QB (B_), which are included in the same cluster, and they are also related to the animals in group AB. These results show that there is a clear differentiation of the animals transmitting a better coat color (A_) compared with the animals transmitting a worse coat color (B_), which is independent of the presence or absence of white marks. This could be explained by the existence of different founders in the different groups. Therefore, the genetic inheritance of both coat traits is independent and could be determined by different genes, although the genetic correlation obtained between both coat traits analyzed (−0.272) confirms the existence of relationship between them. Perhaps the different phenotypes for coat color can be explained by the influence of modifying genes having only a minor effect, which can be cumulative [16]. In this context, the inheritance of the quality of black coat color can have a polygenic component, while the inheritance of white marks is independent and could be determined by genetic and intrauterine effects [32,33,36,49] according to the reviewed bibliography.

## 4. Conclusions

The heritability values obtained for the morpho-functional traits related to coat color in PRMe horses indicate that selection is feasible, and that these traits could be improved in the official Breeding Program. The genetic correlations of coat color traits with linear morpho-functional traits evidence the existence of genetic and physiological mechanisms controlling them, showing that selection for one of these traits could have an influence on (e.g., increase or decrease, according to the sign and value) the other traits of interest. The existence of pleiotropic effects between the coat color traits and some morpho-functional linear traits could be also suspected because of the high genetic correlations obtained between some morpho-functional and coat color analyzed traits. The study of Mahalanobis distances showed that the inheritance of the quality of coat color could be linked to the genetic group and determined by modifying genes. In this sense, a genomics analysis could have an interesting contribution to confirming our results in the future.

## Figures and Tables

**Table 1:** Description of the 46 linear morpho-functional traits analyzed in Pura Raza Menorquina Horses.

	Trait		Class			Trait		Class	
			1	7				1	7
**Color**	**1**	Quality of black coat	Pale	Very dark	**Morphological traits**	**24**	Chest width	Very narrow	Very wide
	**2**	Percentage of white marks	Absence	Excess		**25**	Thorax width	Very narrow	Very wide
**Morphological traits**	**3**	Head width	Very narrow	Very wide		**26**	Forearm length	Very short	Very long
	**4**	Head length	Very short	Very long		**27**	Cannon bone length	Very short	Very long
	**5**	Head depth	Shallow	Very deep		**28**	Fore knee perimeter	Very narrow	Very wide
	**6**	Head profile	Concave	Convex		**29**	Cannon bone perimeter	Very narrow	Very wide
	**7**	Head expression	Not much	Very much		**30**	Forelimb side view	Camped under	Camped out
	**8**	Neck length	Very short	Very long		**31**	Fore hoof-pastern axis side view	Horizontal	Vertical
	**9**	Head-neck junction	Very covered	Very marked		**32**	Fore-hoof slope	Horizontal	Vertical
	**10**	Upper neck line	Concave	Convex		**33**	Form of hoof	Cylindrical	Bell-shaped
	**11**	Lower neck line	Concave	Convex		**34**	Fore-hoof front view	Toe-in	Toe-out
	**12**	Height of withers	Very short	Very high		**35**	Forelimb front view	Closed	Open
	**13**	Shape of withers	Not prominent	Very prominent		**36**	Hock side view	Closed	Open
	**14**	Shoulder length	Very short	Very long		**37**	Hock rear view	Closed	Open
	**15**	Shoulder angle slope	Very horizontal	Very vertical	**Functional traits**	**38**	Walking activity	Very little	Hasty
	**16**	Thorax depth	Shallow	Very deep		**39**	Walking clarity	Very little	Much
	**17**	Back length	Very short	Very long		**40**	Walking amplitude	Very little	Wide
	**18**	Loin length	Very short	Very long		**41**	Walking suppleness	Very little	Much
	**19**	Shape of back-loin line	Very concave	Slightly convex		**42**	Trotting amplitude	Very little	Wide
	**20**	Croup length	Very short	Very long		**43**	Trotting suppleness	Very little	Much
	**21**	Croup angle	Very horizontal	Very vertical		**44**	Trotting impulsion	Very little	Much
	**22**	Buttock length	Very short	Very long		**45**	Trotting equilibrium	Downhill	Uphill
	**23**	Withers-croup equilibrium	Downhill	Uphill		**46**	Trotting suspension	Very little	Much

**Table 2:** Basic statistics of the 46 linear traits analyzed in Pura Raza Menorquina horses.

	Trait	Mean ± s.e.	CV (%)	Range		Trait	Mean ± s.e.	CV (%)	Range
**CT**	**1**	4.80 ± 0.046	26.49	7	**MT**	**24**	5.01 ± 0.046	25.63	7
	**2**	1.51 ± 0.036	64.95	7		**25**	4.83 ± 0.039	22.69	6
**MT**	**3**	5.23 ± 0.042	22.45	7		**26**	4.01 ± 0.027	18.78	6
	**4**	4.80 ± 0.038	21.72	6		**27**	3.55 ± 0.025	19.68	5
	**5**	4.48 ± 0.033	20.30	6		**28**	3.67 ± 0.029	21.80	6
	**6**	4.49 ± 0.032	19.53	7		**29**	3.21 ± 0.025	21.43	5
	**7**	4.82 ± 0.032	18.68	6		**30**	3.50 ± 0.022	17.36	5
	**8**	4.84 ± 0.037	21.27	7		**31**	4.16 ± 0.029	19.10	6
	**9**	3.91 ± 0.040	28.11	7		**32**	3.98 ± 0.027	18.50	5
	**10**	5.16 ± 0.028	15.31	6		**33**	3.42 ± 0.027	21.81	6
	**11**	4.12 ± 0.030	20.52	6		**34**	4.30 ± 0.035	22.38	6
	**12**	4.76 ± 0.043	25.32	6		**35**	4.03 ± 0.024	16.79	6
	**13**	4.40 ± 0.052	32.89	7		**36**	4.40 ± 0.035	22.23	6
	**14**	4.95 ± 0.034	19.10	6		**37**	4.00 ± 0.038	26.51	7
	**15**	3.95 ± 0.042	29.18	7	**FT**	**38**	4.04 ± 0.031	21.42	7
	**16**	4.66 ± 0.037	21.81	6		**39**	4.71 ± 0.038	22.20	6
	**17**	3.99 ± 0.029	19.87	5		**40**	4.35 ± 0.034	21.62	7
	**18**	3.45 ± 0.030	23.77	6		**41**	4.38 ± 0.037	23.19	7
	**19**	3.85 ± 0.030	21.36	7		**42**	4.59 ± 0.036	21.76	7
	**20**	4.23 ± 0.031	20.53	7		**43**	4.33 ± 0.038	24.08	7
	**21**	4.48 ± 0.042	25.77	7		**44**	4.70 ± 0.041	24.02	7
	**22**	3.52 ± 0.037	29.52	7		**45**	4.50 ± 0.037	22.85	6
	**23**	3.89 ± 0.039	28.06	7		**46**	4.52 ± 0.042	25.45	7

CT are coat color traits (1 and 2), MT are morphological traits (3–11 related to head and neck, 12–25 related to body regions and 26–37 related to limbs), and FT are functional traits (38–41 related to walking and 41–46 related to trotting). Traits names are shown in Table 1.

**Table 3:** Additive, permanent environmental and residual variances, and heritability values for the 46 linear traits analyzed in Pura Raza Menorquina horses.

	Tr	σ^2^_u_ (s.d.)	σ^2^_pe_ (s.d.)	σ^2^_e_ (s.d.)	h^2^ (s.d.)		Tr	σ^2^_u_ (s.d.)	σ^2^_pe_ (s.d.)	σ^2^_e_ (s.d.)	h^2^ (s.d.)
**CT**	**1**	0.47 (0.146)	0.32 (0.115)	0.51 (0.004)	0.36 (0.098)	**MT**	**24**	0.41 (0.137)	0.36 (0.114)	0.57 (0.039)	0.30 (0.091)
	**2**	0.22 (0.146)	0.64 (0.125)	0.08 (0.001)	0.23 (0.142)		**25**	0.30 (0.090)	0.19 (0.073)	0.43 (0.030)	0.32 (0.086)
**MT**	**3**	0.36 (0.067)	0.05 (0.039)	0.53 (0.035)	0.38 (0.057)		**26**	0.15 (0.056)	0.14 (0.047)	0.27 (0.019)	0.26 (0.088)
	**4**	0.45 (0.105)	0.12 (0.071)	0.46 (0.031)	0.44 (0.083)		**27**	0.10 (0.035)	0.10 (0.033)	0.28 (0.019)	0.21 (0.068)
	**5**	0.24 (0.055)	0.07 (0.042)	0.47 (0.032)	0.30 (0.062)		**28**	0.11 (0.042)	0.09 (0.038)	0.31 (0.021)	0.21 (0.077)
	**6**	0.43 (0.076)	0.07 (0.046)	0.24 (0.017)	0.58 (0.073)		**29**	0.09 (0.034)	0.10 (0.032)	0.25 (0.018)	0.19 (0.071)
	**7**	0.10 (0.061)	0.16 (0.058)	0.45 (0.031)	0.14 (0.081)		**30**	0.03 (0.025)	0.16 (0.029)	0.18 (0.012)	0.09 (0.065)
	**8**	0.36 (0.105)	0.13 (0.078)	0.47 (0.032)	0.37 (0.094)		**31**	0.14 (0.064)	0.17 (0.055)	0.29 (0.020)	0.23 (0.098)
	**9**	0.31 (0.105)	0.19 (0.089)	0.65 (0.045)	0.27 (0.082)		**32**	0.13 (0.046)	0.10 (0.041)	0.31 (0.021)	0.24 (0.079)
	**10**	0.15 (0.057)	0.21 (0.049)	0.19 (0.013)	0.28 (0.093)		**33**	0.15 (0.066)	0.16 (0.055)	0.27 (0.019)	0.26 (0.104)
	**11**	0.15 (0.080)	0.14 (0.066)	0.37 (0.026)	0.23 (0.111)		**34**	0.26 (0.116)	0.46 (0.101)	0.25 (0.017)	0.26 (0.109)
	**12**	0.27 (0.132)	0.51 (0.122)	0.59 (0.040)	0.19 (0.090)		**35**	0.05 (0.042)	0.22 (0.042)	0.21 (0.014)	0.11 (0.083)
	**13**	0.37 (0.151)	0.58 (0.142)	0.80 (0.055)	0.21 (0.079)		**36**	0.09 (0.072)	0.49 (0.081)	0.41 (0.028)	0.10 (0.070)
	**14**	0.28 (0.077)	0.11 (0.059)	0.46 (0.031)	0.33 (0.079)		**37**	0.23 (0.113)	0.51 (0.106)	0.37 (0.026)	0.20 (0.094)
	**15**	0.13 (0.082)	0.21 (0.080)	0.73 (0.050)	0.12 (0.074)	**FT**	**38**	0.06 (0.050)	0.26 (0.054)	0.35 (0.024)	0.09 (0.070)
	**16**	0.32 (0.065)	0.06 (0.043)	0.39 (0.026)	0.41 (0.069)		**39**	0.09 (0.065)	0.35 (0.074)	0.56 (0.039)	0.09 (0.063)
	**17**	0.05 (0.036)	0.13 (0.041)	0.41 (0.028)	0.09 (0.059)		**40**	0.31 (0.119)	0.23 (0.093)	0.39 (0.027)	0.33 (0.113)
	**18**	0.09 (0.034)	0.04 (0.029)	0.41 (0.027)	0.16 (0.060)		**41**	0.33 (0.118)	0.19 (0.093)	0.51 (0.035)	0.32 (0.102)
	**19**	0.07 (0.038)	0.18 (0.041)	0.30 (0.021)	0.12 (0.065)		**42**	0.21 (0.089)	0.34 (0.080)	0.34 (0.024)	0.23 (0.092)
	**20**	0.17 (0.067)	0.13 (0.058)	0.46 (0.032)	0.23 (0.081)		**43**	0.20 (0.087)	0.27 (0.077)	0.43 (0.030)	0.22 (0.088)
	**21**	0.25 (0.099)	0.35 (0.092)	0.57 (0.039)	0.21 (0.078)		**44**	0.30 (0.129)	0.37 (0.108)	0.42 (0.029)	0.28 (0.106)
	**22**	0.08 (0.054)	0.19 (0.058)	0.55 (0.038)	0.10 (0.063)		**45**	0.25 (0.096)	0.23 (0.082)	0.48 (0.033)	0.26 (0.090)
	**23**	0.26 (0.119)	0.43 (0.109)	0.54 (0.038)	0.21 (0.090)		**46**	0.23 (0.129)	0.48 (0.116)	0.44 (0.031)	0.20 (0.103)

σ^2^_u_ is additive genetic variance; σ^2^_pe_ is permanent environmental variance; σ^2^_e_ is residual variance; h² is heritability; s.d. is standard deviation; CT are coat color traits (1 and 2); MT are morphological traits (3–11 related to head and neck, 12–25 related to body regions and 26–37 related to limbs) and FT are functional traits (38–41 related to walking and 41–46 related to trotting). Trait names are shown in Table 1.

**Table 4:** Genetic correlations (rg) obtained for the traits related with coat color (1: quality of black coat color and 2: white marks) and the 44 morpho-functional traits analyzed in Pura Raza Menorquina horses.

	Trait	rg1 (s.d.)	rg2 (s.d.)		Trait	rg1 (s.d.)	rg2 (s.d.)
**MT**	**3**	−0.114 (0.209)	−0.065 (0.410)	**MT**	**25**	0.099 (0.243)	−0.155 (0.465)
	**4**	0.186 (0.206)	−0.443 (0.372)		**26**	0.015 (0.268)	−0.177 (0.504)
	**5**	−0.090 (0.224)	0.295 (0.377)		**27**	0.047 (0.275)	0.084 (0.479)
	**6**	0.609 (0.184)	−0.204 (0.382)		**28**	−0.349 (0.430)	0.617 (0.402)
	**7**	0.154 (0.355)	−0.030 (0.584)		**29**	−0.738 (0.225)	0.448 (0.444)
	**8**	0.337 (0.234)	−0.404 (0.399)		**30**	−0.170 (0.270)	−0.175 (0.484)
	**9**	−0.325 (0.270)	0.074 (0.503)		**31**	0.366 (0.255)	−0.269 (0.524)
	**10**	0.816 (0.171)	−0.434 (0.393)		**32**	0.226 (0.272)	0.069 (0.505)
	**11**	−0.441 (0.302)	0.227 (0.534)		**33**	0.094 (0.445)	−0.807 (0.235)
	**12**	−0.493 (0.323)	0.469 (0435)		**34**	0.311 (0.418)	−0.603 (0.414)
	**13**	−0.767 (0.216)	0.577 (0.372)		**35**	0.025 (0.325)	0.076 (0.560)
	**14**	0.187 (0.219)	0.224 (0.430)		**36**	0.310 (0.428)	0.098 (0.598)
	**15**	−0.419 (0.327)	0.638 (0.365)		**37**	0.185 (0.398)	−0.110 (0.574)
	**16**	0.269 (0.203)	0.329 (0.349)	**FT**	**38**	0.123 (0.244)	0.396 (0.420)
	**17**	0.618 (0.298)	0.336 (0.529)		**39**	0.318 (0.231)	−0.574 (0.316)
	**18**	0.292 (0.299)	−0.045 (0.573)		**40**	0.325 (0.247)	−0.078 (0.492)
	**19**	−0.224 (0.340)	−0.379 (0.484)		**41**	0.243 (0.257)	−0.206 (0.475)
	**20**	0.307 (0.263)	−0.295 (0.479)		**42**	0.376 (0.226)	−0.115 (0.502)
	**21**	−0.459 (0.296)	0.139 (0.483)		**43**	0.224 (0.254)	−0.199 (0.482)
	**22**	0.229 (0.346)	−0.576 (0.405)		**44**	0.241 (0.296)	−0.379 (0.498)
	**23**	−0.371 (0.320)	0.159 (0.518)		**45**	0.099 (0.243)	−0.155 (0.465)
	**24**	0.411 (0.227)	0.014 (0.446)		**46**	0.015 (0.268)	−0.177 (0.504)

MT are morphological traits (3–11 related to head and neck, 12–25 related to body regions and 26–37 related to limbs) and FT are functional traits (38–41 related to walking and 41–46 related to tro-tting). rg1 is the genetic correlation obtained for the quality of the black coat color -QB- and rg2 is the genetic correlation obtained for the white marks -WM-; s.d. is standard deviation; Traits names are shown in Table 1.

## Data Availability

Restrictions apply to the availability of these data. Data were obtained from Asociación de Criadores y Propietarios de Caballos de Raza Menorquina (ACPCRMe) and are available from the corresponding author with the permission of ACPCRMe.

## Supplementary Materials

The following supporting information can be downloaded at: https://www.mdpi.com/article/10.3390/ani12182319/s1, Table S1: Analysis of the influence of the different effects on 46 morpho-functional linear traits analyzed in Pura Raza Menorquina horses using a univariate GeneralLinear Model analysis of variance with a permanent environmental effect (*p* values).
