# Supplementary material for: Morpho-Functional Traits in Pura Raza Menorquina Horses: Genetic Parameters and Relationship with Coat Color Variables
Source: Animals (Basel). 2022 Sep 7;12(18):2319. doi: 10.3390/ani12182319 (PMC9495247; doi:10.3390/ani12182319)
Supplement: Supplementary file 1 [file animals-12-02319-s001.zip › animals-1765213-supplementary.pdf]

Supplementary Table S1. Analysis of the influence of the different effects on 46 morpho-functional linear traits analyzed in Pura Raza Menorquina horses, using a univariate General Linear Model analysis of variance with permanent environmental effect (p values).

	Trait	S	A-S	QB	WM	Ag	PE	R^2	RMSE
CT	1	<0.0001	0.898	-	<0.0001	0.023	<0.0001	0.584	1.988
	2	0.095	0.852	0.074	-	0.419	<0.0001	0.663	1.093
MT	3	<0.0001	<0.0001	0.405	0.002	<0.0001	<0.0001	0.820	0.674
	4	0.001	<0.0001	0.292	0.987	0.998	<0.0001	0.770	0.676
	5	<0.0001	<0.0001	0.156	0.484	0.803	<0.0001	0.693	0.681
	6	<0.0001	<0.0001	0.866	0.918	0.847	<0.0001	0.828	0.492
	7	0.000	<0.0001	0.759	0.260	0.021	<0.0001	0.711	0.655
	8	0.049	<0.0001	<0.0001	0.048	0.063	0.388	0.712	0.829
	9	<0.0001	0.145	0.085	0.001	0.109	<0.0001	0.670	0.928
	10	<0.0001	<0.0001	0.019	0.615	0.431	<0.0001	0.752	0.633
	11	0.001	<0.0001	0.455	0.581	0.400	<0.0001	0.731	0.594
	12	0.138	<0.0001	0.186	0.304	<0.0001	<0.0001	0.791	0.746
	13	<0.0001	<0.0001	0.640	0.086	0.068	<0.0001	0.793	0.892
	14	0.549	<0.0001	0.845	0.031	<0.0001	<0.0001	0.740	0.651
	15	<0.0001	<0.0001	0.707	0.536	0.276	<0.0001	0.617	1.110
	16	<0.0001	0.001	0.645	0.517	0.007	<0.0001	0.797	0.620
	17	0.000	<0.0001	0.979	0.587	0.002	<0.0001	0.662	0.625
	18	0.000	<0.0001	0.922	0.230	0.003	<0.0001	0.656	0.742
	19	0.095	<0.0001	0.741	0.700	0.003	<0.0001	0.760	0.546
	20	0.141	<0.0001	0.389	0.073	0.001	<0.0001	0.677	0.762
	21	<0.0001	<0.0001	0.830	0.624	<0.0001	<0.0001	0.789	0.718
	22	0.011	<0.0001	0.446	0.537	0.088	<0.0001	0.613	1.024
	23	0.507	0.017	0.740	0.343	0.443	<0.0001	0.713	0.861
MT	24	0.004	<0.0001	0.940	0.082	<0.0001	<0.0001	0.821	0.734
	25	<0.0001	<0.0001	0.245	0.796	<0.0001	<0.0001	0.751	0.812
	26	0.665	<0.0001	0.086	0.155	0.011	<0.0001	0.751	0.509
	27	0.362	<0.0001	0.000	0.238	0.610	0.020	0.640	0.675
	28	<0.0001	0.001	0.439	0.087	0.011	<0.0001	0.611	0.948
	29	<0.0001	<0.0001	0.225	0.003	<0.0001	<0.0001	0.639	0.664
	30	0.040	0.000	0.137	0.869	0.342	<0.0001	0.742	0.418
	31	<0.0001	<0.0001	0.404	0.002	0.438	<0.0001	0.761	0.525
	32	0.002	0.028	0.880	0.961	0.574	<0.0001	0.694	0.551
	33	0.829	0.049	0.078	0.979	0.212	<0.0001	0.545	0.980
	34	<0.0001	0.001	0.001	0.576	0.212	<0.0001	0.861	0.486
	35	0.227	0.552	0.892	0.250	0.723	<0.0001	0.676	0.634
	36	0.579	0.004	0.178	0.714	0.439	<0.0001	0.706	0.800
	37	0.043	0.682	0.558	0.901	0.718	<0.0001	0.686	1.011
FT	38	<0.0001	0.000	0.273	0.598	0.156	<0.0001	0.854	0.780
	39	<0.0001	<0.0001	0.173	0.306	0.003	<0.0001	0.840	0.902
	40	0.008	0.008	0.116	0.218	0.001	<0.0001	0.858	0.803
	41	0.003	<0.0001	0.960	0.065	0.350	<0.0001	0.840	0.878
	42	0.057	<0.0001	0.344	0.560	0.394	<0.0001	0.872	0.790
	43	0.994	<0.0001	0.617	0.193	0.399	<0.0001	0.836	0.932
	44	0.094	<0.0001	0.767	0.262	0.544	<0.0001	0.849	0.938
	45	0.530	<0.0001	0.777	0.437	0.072	<0.0001	0.798	1.078
	46	0.001	<0.0001	0.426	0.122	0.408	<0.0001	0.828	1.039

CT are coat color traits (1 and 2), MT are morphological traits (3-11 related to head and neck, 12-25 related to body regions and 26-37 related to limbs), and FT are functional traits (38-41 related to walk and 41-46 related to trot). Traits names are shown in table 1. S is the effect of sex, A-S is the effect of the appraiser-season; QB is the effect of coat quality; WM is the effect of percentage of white marks; Ag is the effect of the age (linear covariate); PE is the permanent environmental effect; R2 is the coefficient of determination and RMSE is the Root mean squared error. Trait 1 is not included in the analysis of the effect of QB and trait 2 is not included in the analysis of the effect of WM.
